# The importance of epistemic intentions in ascription of responsibility

**DOI:** 10.1038/s41598-023-50961-0

**Published:** 2024-01-12

**Authors:** Katarina M. Kovacevic, Francesca Bonalumi, Christophe Heintz

**Affiliations:** 1 Department of Cognitive Science, Central European University, Vienna, 1100 Austria; 2 School of Social Sciences and Technology, Technical University Munich, Munich, Germany

**Keywords:** Human behaviour, Decision

## Abstract

We investigate how people ascribe responsibility to an agent who caused a bad outcome but did not know he would. The psychological processes for making such judgments, we argue, involve finding a counterfactual in which some minimally benevolent intention initiates a course of events that leads to a better outcome than the actual one. We hypothesize that such counterfactuals can include, when relevant, epistemic intentions. With four vignette studies, we show that people consider epistemic intentions when ascribing responsibility for a bad outcome. We further investigate which epistemic intentions people are likely to consider when building counterfactuals for responsibility ascription. We find that, when an agent did not predict a bad outcome, people ascribe responsibility depending on the reasons behind the agents’ lack of knowledge. People judge agents responsible for the bad outcome they caused when they could have easily predicted the consequences of their actions but did not care to acquire the relevant information. However, when this information was hard to acquire, people are less likely to judge them responsible.

## Introduction

Rose was rushing to go to the dentist, and she parked in a spot that was reserved for people with disabilities. She did not know that, but she did not check either. As a result, Philip could not park in his reserved spot and missed his weekly therapy session. Is Rose responsible for that regrettable outcome? Is she responsible even though she did not know? What if Rose had very good reasons to believe that the spot was not reserved? What if checking the availability of the spot involved waiting for the parking attendant for a long time and thus missing an important meeting herself? People ascribe responsibility to agents who made a choice knowing that it would have negative consequences on others, but judgments about responsibility vary greatly when the choice is made without such knowledge^1–3^. What underlying cognitive processes produce these judgments?

In our account, people ascribe responsibility for bringing about a negative outcome not only to agents who predicted such an outcome but also to agents who did not predict it. In such cases, people ascribe responsibility by constructing counterfactuals, i.e, by reasoning about what could have been done to avoid the bad outcome. Counterfactuals are imagined alternatives to real events^4^. People conceive counterfactuals not only when developing causal explanations^5,6^, but also when ascribing blame^7,8^ and responsibility^9–11^. However, previous work that examined the role of counterfactual reasoning in moral judgments focused mostly on agents’ actions^10–13^ and agents’ epistemic states^14–16^. Less research emphasizes the role of counterfactual reasoning about agents’ intentions^16,17^.

People answer questions about others’ responsibility with the goal of deciding whom to blame or praise, and, more generally, whom to hold accountable^18–20^. With these moral goals in mind, agents’ intentions become especially relevant^21,22^. One reason why intentions are especially relevant when formulating such judgments is that they are key evidence of the agents’ prosocial preferences - and, in particular, about how much they cared for others when making their choice^23,24^. For instance, an agent who does not throw an emergency buoy at a drowning man just because they do not want to is deemed responsible for the death of that man. An agent who does want to throw a buoy but does not find one is instead not deemed responsible unless there is another option for saving the drowning man. Furthermore, an agent who chooses not to put their own life at risk to save a drawing man is usually *not* deemed responsible, while an agent who chooses not to get their clothes wet to save a drowning man usually is. Counterfactuals are relevant for responsibility judgments to the extent that they reveal whether a person with minimal concern for others could have, with the right intentions, avoided the bad outcome.

**Figure 1:**
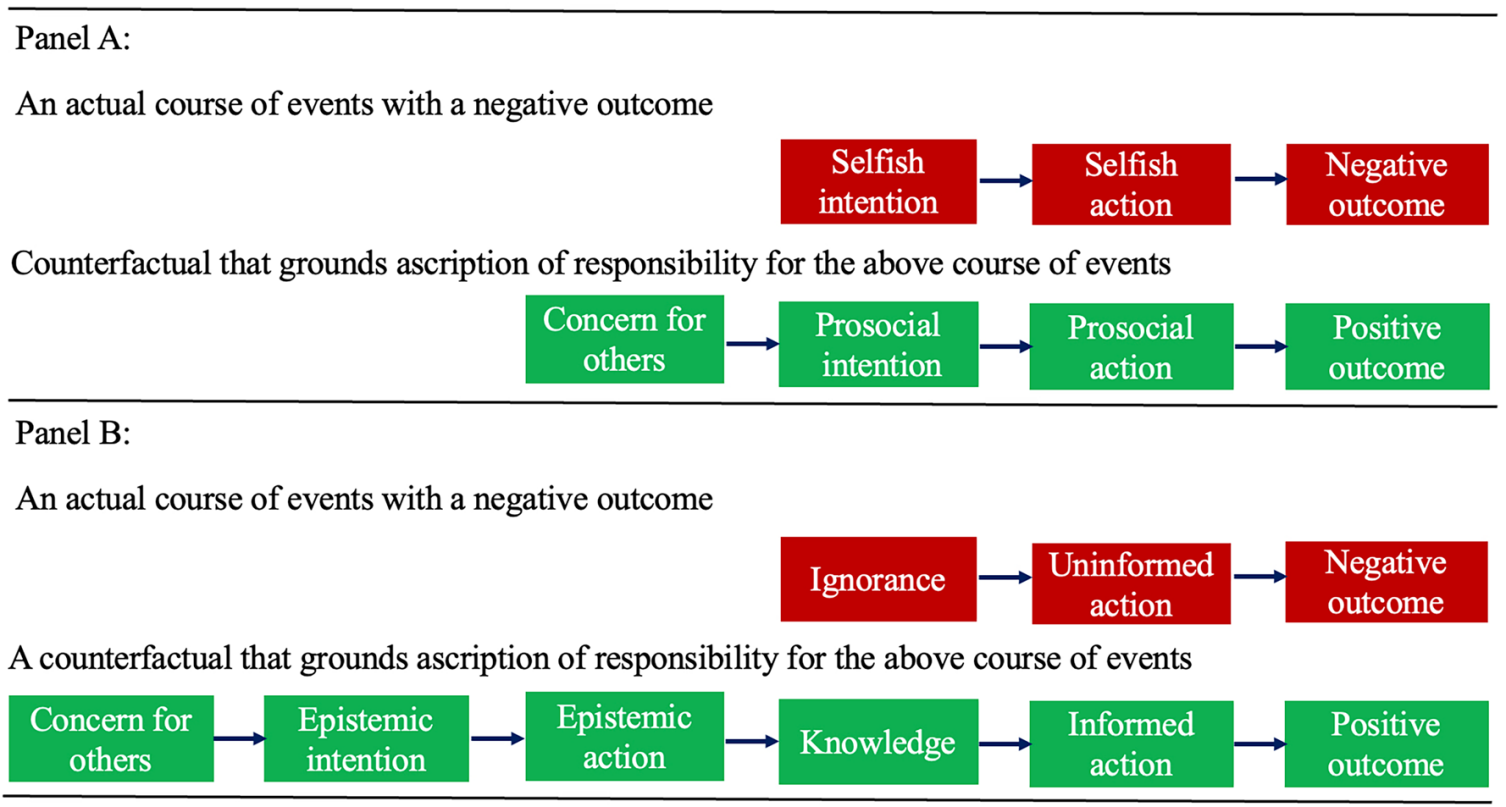
Ascription of responsibility involves thinking of counterfactual in which alternative intentions - based on sufficient concern for others - lead to alternative outcomes. Panel A illustrates counterfactual thinking when the alternative intention is directly related to the proximal act of causing the negative outcome. Panel B illustrates counterfactual thinking when the alternative intention is of an epistemic nature and has a more distal effect on the outcome at stake.

These considerations about the relevance of intentions for ascribing responsibility apply also to intentions that are specifically epistemic. Epistemic intentions are intentions that drive actions meant to acquire information^25^. Counterfactuals in which the assessed agent has an alternative epistemic intention are especially relevant when the assessed agent did not predict the bad consequences of their choice. We reasoned that people ascribe responsibility if they can think of a counterfactual in which the assessed agent has an epistemic intention that initiates a causal chain of events that eventually prevents the negative outcome from happening (See Fig. 1).

Epistemic intentions have a more distal causal role in bringing about the target outcome. Still, we hold that people making judgments about responsibility do conceive counterfactuals with alternative epistemic intentions. In fact, people do think of counterfactuals related to the initial instances in a specific causal sequence^26^, and counterfactuals can include changing the state of knowledge^15^. Counterfactuals that form the basis for ascribing responsibility might take the form of a causal chain of events that is initiated by a counterfactual epistemic intention. The intention causes an epistemic action, which in turn informs a choice, which avoids the bad outcome. The counterfactual epistemic intention reveals whether the assessed agent acted with moral concern for others, or not^27^. The reasoning is that, if the agent failed to have an epistemic intention that would have led to a better outcome for others, it is because that agent cared too little about their welfare. However, not all alternative epistemic intentions and the counterfactual chains of events they initiate are considered bases for responsibility ascription. We do not expect agents to pay unreasonable costs to acquire relevant information, and we do not expect them to intend epistemic actions that have very little chance to make a difference. Expectations about epistemic actions are, here, similar to expectations about non-epistemic action: we do not expect people to put their lives at risk to save a drowning man, for instance.

We hypothesize that people ascribe responsibility to an agent who did not predict that their choice would have bad consequences if people think of a counterfactual that has the following properties:

- The assessed agent has an alternative epistemic intention that would lead to an intended epistemic action.
- The intended epistemic action is not too costly for the assessed agent and it is expected to be sufficiently informative (it is sufficiently likely to lead the agent to change their mind and make a different choice).
- The chain of events that causally follows from this alternative intention leads to an outcome that is better than the actual one.

We tested this hypothesis by running a series of preregistered studies in which we asked participants to read various vignettes that described events that eventually led to a bad outcome. Participants were requested to state whether they agreed that an agent, the main protagonist of the vignette, was responsible for a given outcome. The vignettes described situations in which the protagonists could, or could not predict the consequences of their actions.

In the vignettes, we manipulated whether the agents knew about the potential consequences of their actions and the reasons behind their ignorance. The reasons for ignoring relevant information depended on whether the agent had the opportunity to learn (Study 1a), or the intention to learn (Study 1b), whether it was easy or difficult to acquire the information (Study 2a), and if the available epistemic action was sufficiently informative or not (Study 2b).

Our goal was to show that, when ascribing responsibility to agents who did not predict the consequences of their choice, the cognitive processes include:

1. Thinking of counterfactuals that start with alternative epistemic intentions. Consequently:
  1. Responsibility ascription depends on the opportunity that the agent had to learn the relevant information (Study 1a)
  2. Responsibility ascription depends on the evidence about the target agent’s epistemic intentions, and whether or not the epistemic action was taken (Study 1b).
2. Thinking of alternative epistemic intentions that have sufficient expected value. Consequently:
  H2a Responsibility ascription depends on the cost of the intended epistemic action (Study 2a)
  H2b Responsibility ascription depends on the envisaged benefit of the intended epistemic action (Study 2b).

## Data analysis

To test our hypotheses, we ran a series of cumulative link mixed models to assess the effect of the experimental conditions on participants’ ratings. The main models always included condition as fixed effect, and condition nested within vignettes as random effect. Study 1a only also featured participant ID as an additional random factor, to account for repeated measures. The null models included the random effects only. Model comparisons were done using likelihood ratio tests^28^. Statistics were done using R version 4.2.3^29^ and the package ‘ordinal’^30^. Correlation tests were done in Jamovi version 2.3^31^. Following reviewers’ suggestions, some of the final analyses presented in the manuscript deviate from the preregistered version, with largely consistent results (See Supplementary Note online https://osf.io/y9gxj/).

## Results

### Study 1a

Study 1a tests the hypothesis that the ascription of responsibility depends both on the assessment of agent’s epistemic states and on their epistemic actions. In more detail, we test whether responsibility ascription depends on the opportunity to learn the relevant information (H1a). If there is an opportunity to learn, then there is a counterfactual in which the intention to seize that opportunity gives a counterfactual in which the bad outcome is avoided. In turn, this counterfactual constitutes a basis for ascribing responsibility. By contrast, if there was no such opportunity, then the intention cannot be acted upon and the above counterfactual does not lead to avoiding the bad outcome. There is no basis for ascribing responsibility. To test this, we designed three different scenarios and across these scenarios, we compared conditions in which we manipulated these epistemic factors: in the *Knowledge* condition the agent knows relevant information (i.e., that their action will bring about a bad outcome); in the *Full Ignorance* condition the agent does not have such knowledge; and in the *Willful Ignorance* condition the agent does not have knowledge about the relevant information, but has the opportunity to take an epistemic action to acquire such knowledge and willfully does not take it. We measured participants’ responsibility judgments by prompting them to rate on a Likert scale to what extent they agreed that the agent was responsible for the bad outcome (Responsibility Question). We predicted that responsibility is ascribed more often when an agent brought about an undesirable outcome as a consequence of their willful ignorance in comparison to the case when the agent fully unknowingly brought about the same outcome. We further expected that responsibility is ascribed more often when the agent knowingly brought about the bad outcome.

**Figure 2:**
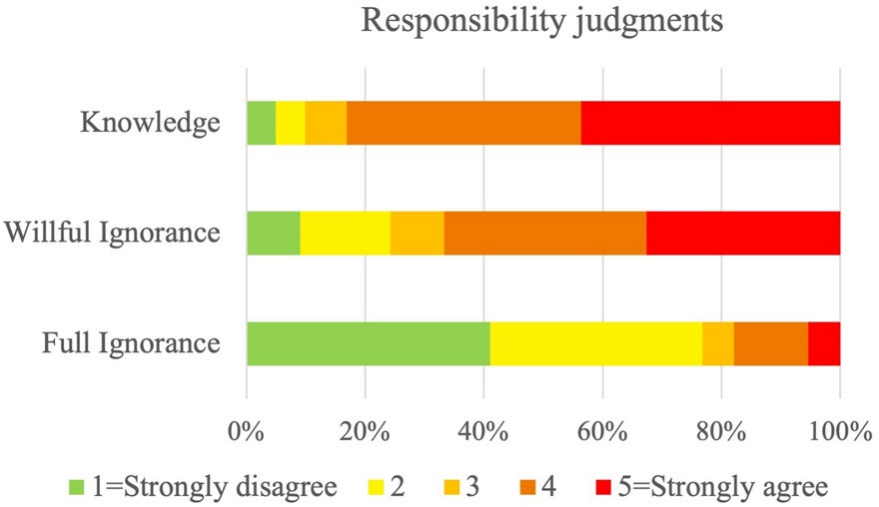
Distributions of responsibility judgments across different epistemic status conditions: In the *Full Ignorance* condition, participants read scenarios in which an agent could not know what bad outcome their actions would have on others. In the *Willful Ignorance* condition, the agent had the opportunity to acquire the relevant information but chose not to. In the *Knowledge condition*, the agent knew about the possible consequences of their action. Participants answered the question: “To what extent do you agree with the statement: ‘[Agent] is responsible for [outcome]’?”. Answers were given on a five-point scale from ‘Strongly disagree’, to ‘Strongly agree’. Responsibility was ascribed significantly less often in the *Full Ignorance* condition than in the *Willful Ignorance* and the *Knowledge* condition.

To test H1a, we built a cumulative link mixed model and compared it with a null model using a likelihood ratio test. The model showed a better fit of the data compared to the null model, $\chi ^{2}(2) = 8.22$, $p = 0.016$ (See Supplementary Table S5a online). Consistent with our prediction, the model indicates that participants’ responses differed significantly across conditions. The results of the pairwise comparisons (with p-values adjusted with Bonferroni correction) indicated that the *Full Ignorance* condition differs significantly from the *Willful Ignorance* condition ($\beta$ = − 3.05, SE = 0.79, *z* = − 3.84, $p < 0.001$) and Knowledge condition ($\beta$ = − 3.72, SE = 0.62, *z* = − 5.98, $p < 0.001$) so that participants were less likely to ascribe responsibility to fully ignorant agents than in the other conditions. Willfully ignorant agents were not significantly more likely to be given lower ratings compared to participants in the *Knowledge* condition ($\beta$ = − 0.68, SE = 0.47, *z* = − 1.43, $p = 0.454$). See Fig. 2.

Our findings corroborate our first hypothesis. Participants were sensitive to the epistemic status of the agent. First, beliefs mattered: participants were more willing to ascribe responsibility to an agent who had knowingly brought about a bad outcome than to the agents who did not have this knowledge. Second, epistemic actions mattered: participants were more willing to ascribe responsibility when an agent brought about an undesirable outcome as a consequence of their lack of epistemic action in comparison to cases when the agent had no opportunity to take an epistemic action.

In this study, we did not explicitly state the agent’s epistemic intentions in the scenario descriptions. We stated only the circumstances and the agent’s decision given those circumstances, and it was left to participants to infer, especially in the Willful ignorance condition, whether the agent lacked prosocial intentions or not. In the next study, we disentangle further the role of epistemic intention, action, and state. For that purpose, we created scenarios in which we provided explicit information about the agent’s epistemic intentions.

### Study 1b

Study 1b tests the hypothesis that when assessing the responsibility of an agent, people will be sensitive to evidence about the target agent’s epistemic intentions that are given verbally and in an explicit way (H1b). For that purpose, we designed three different scenarios, and across these scenarios, we compared conditions in which we manipulated the agent’s epistemic intention and epistemic action. In the *Willful Ignorance* condition the agent does not want to learn the relevant information (they have no prosocial epistemic intention) and they do not take the epistemic action. This condition is the same as in Study 1a, but some intentions are explicitly stated. In the *Circumstantial Ignorance* condition, the agent has a prosocial epistemic intention, but the circumstances make the associated epistemic action impossible. In the *Misinformed Ignorance* condition, the agent has a prosocial epistemic intention and takes the associated epistemic action, but gets misinformed. In all of the three conditions, the agent’s behavior produces negative consequences. We measured participants’ responsibility judgments by prompting them to rate on a Likert scale to what extent they agreed that the agent was responsible for the bad outcome (Responsibility Question). In addition, participants were prompted to rate on a Likert scale how much the agent cared to learn the relevant information (Care Question). We predicted that participants would be less likely to ascribe responsibility in the *Misinformed* *Ignorance* condition (i.e. when agents are described as having had a prosocial epistemic intention and took the epistemic action that led to wrong information) than in the *Willful* *Ignorance* condition (i.e., when agents did not show any prosocial epistemic intention nor took any epistemic action). We also predicted that participants would be significantly less likely to ascribe responsibility to those agents in the *Circumstantial* *Ignorance* condition, who showed intention to take the prosocial epistemic action if taking the action was possible, than in the *Willful* *Ignorance* condition where no prosocial epistemic intention was present. We furthermore predicted that the more agents are perceived as they cared to learn the relevant information, the less likely they will be judged responsible.

**Figure 3:**
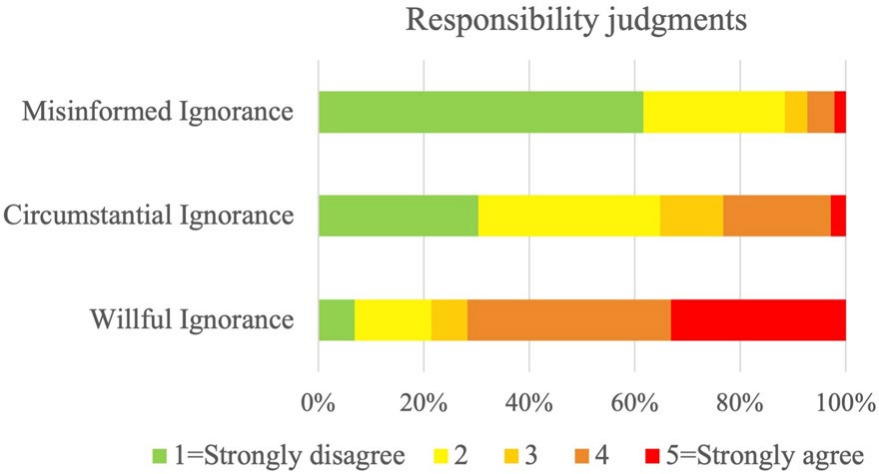
Distributions of responsibility judgments across three different conditions: In the *Willful Ignorance* condition, participants read scenarios in which an agent did not want to know what bad outcome their actions would have on others, and they did not take the epistemic action. In the *Circumstantial Ignorance* condition, the agent wanted to acquire the relevant information, but it was impossible to take the epistemic action. In the *Misinformed Ignorance* condition, the agent wanted to know about the possible consequences of their action and took the epistemic action, but it led to the wrong information. Participants answered the question: “To what extent do you agree with the statement: ‘[Agent] is responsible for [outcome]’?”. Answers were given on a five-point scale from ‘Strongly disagree’, to ‘Strongly agree’. Responsibility was ascribed significantly more often in the *Willful Ignorance* condition than in the *Circumstantial Ignorance* condition, or *Misinformed Ignorance* condition.

To test H1b, we built a cumulative link mixed model and compared it with a null model using a likelihood ratio test. The model showed a better fit of the data $\chi ^{2}(2) = 9.64$, $p = .008$ (See Supplementary Table S6a online). Consistent with our prediction, the model indicates that participants’ responses differed significantly across conditions. The results of the pairwise comparisons indicated that the *Willful Ignorance* condition differed significantly from the *Circumstantial Ignorance* condition ($\beta$ = 2.15, SE = 0.30, *z* = 7.12, $p < .001$) and *Misinformed Ignorance* condition ($\beta$ = 3.50, SE = 0.46, *z* = 7.61, $p < .001$); participants were more likely to ascribe responsibility to willfully ignorant agents. Finally, the *Circumstantial Ignorance* condition also differed significantly from the *Misinformed Ignorance* condition ($\beta$ = 1.36, SE = 0.30, *z* = 4.47, $p < .001$), so participants were more likely to ascribe responsibility to circumstantially ignorant agents. See Fig. 3.

Finally, we ran a Spearman rank correlation test. As predicted, the test showed a significant strong negative relationship between Responsibility judgments and Care, $r_s (423) = -0.681$, $p < 0.001$.

Our findings corroborated our hypotheses: participants were sensitive to the evidence about the target agent’s epistemic intentions that was given explicitly. Participants ascribed responsibility more often to agents who did not have prosocial epistemic intentions, than to agents who had a prosocial epistemic intentions, regardless of whether epistemic action was taken or not.

Furthermore, participants’ responsibility judgments were related to their perception of the agent’s care for learning.

### Study 2a

In our previous experimental manipulations, agents who willingly chose to not acquire relevant information had the opportunity to easily do so (*Willful Ignorance* condition). This choice reveals to the observer that the agents were little concerned about the welfare of others-so little that they did not bother to check what consequences their actions might have had. However, choosing not to acquire the relevant information might be motivated by the cost of the epistemic action that must be performed to acquire this information. When the cost of the epistemic action is high enough, the observer has no evidence that the agent had too little concern for others’ welfare: the agent might be concerned by the welfare of others, but the cost of the epistemic action outweighs the potential benefits for others. In that case, would the observer ascribe responsibility to the agent? This is what we test in Study 2a.

In Study 2a we test the hypothesis that, when ascribing responsibility, people weigh the agents’ costs for performing the epistemic action (H2a). We thus presented scenarios to participants that were similar to those in Study 1, except that we manipulated the costs for the agents to perform the epistemic action. We implemented such costs in terms of effort to acquire the relevant information: in the *High Effort* condition, the agent has an opportunity to acquire the information by putting a degree of time and effort into this inquiry; in the *Low Effort* condition, the agent has an opportunity to acquire the information with ease. We again measured participants’ responsibility judgments by prompting them to rate on a Likert scale to what extent they thought the agent was responsible for the bad outcome. We also measured their understanding of the agent’s costs for performing the epistemic action by prompting them to rate on a Likert scale the effort required by the agent to acquire knowledge (Perceived Effort Question). We predicted that responsibility would be ascribed less often to agents who needed to put a high degree of effort into taking an epistemic action. We further predicted that participants’ responsibility judgments would correlate with their perceived effort judgments.

We first conducted a Mann-Whitney test to ensure that the manipulation worked as expected and that the effort required to undertake the epistemic action was perceived differently in the two conditions. The test showed a significant difference between conditions, $U = 933$, $p < 0.001$, $r_{rb}= 0.913$; participants judged the epistemic action to require effort more frequently in the *High Effort* condition than in the *Low Effort* condition.

**Figure 4:**
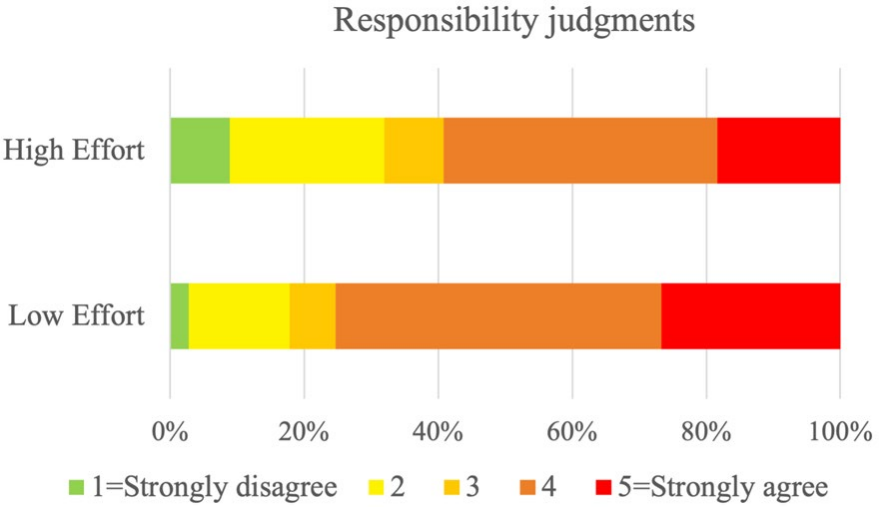
Distributions of responsibility judgments when epistemic actions require low effort or high effort. In the *Low Effort* condition, participants read scenarios in which an agent could easily acquire information about the consequences of their action on others. In the *High Effort* condition, this same epistemic action requested significantly more effort. Participants answered the question: “To what extent do you agree with the statement: ‘[Agent] is responsible for [outcome]’?”. Answers were given on a five-point scale from ‘Strongly disagree’, to ‘Strongly agree’. Responsibility was ascribed significantly more often in the *Low Effort* condition than in the *High Effort* condition.

To test H2a, we ran a cumulative link mixed model to assess the effect of the experimental condition on participants’ ratings of responsibility ascription. We compared our model with a null model using a likelihood ratio test, and the model showed a better fit of the data compared to the null model, $\chi ^{2}(1) = 5.19$ , $p = .023$ (See Supplementary Table S7a online). Consistent with our prediction, the results of the full model indicated that participants in the High Effort condition were 1.92 times more likely to give lower ratings compared to participants in the Low Effort condition, $\beta = -0.65, SE = 0.23$, $\textit{z} = -2.88$. See Fig. 4.

Finally, we ran a Spearman rank correlation test. As predicted, the test showed a significant albeit weak negative correlation between Responsibility judgments and Perceived Effort judgments, $r_s (291) = -0.305$, $p < 0.001$.

Our findings confirmed our hypotheses: participants were sensitive to the effort required by the agent to take an epistemic action. Participants ascribed more responsibility to an agent who had an easy opportunity to acquire knowledge (i.e., by putting in a low degree of effort) and did not, than to an agent who had to put some effort into acquiring the same knowledge. Furthermore, participants’ responsibility judgments were related to their perception of such effort: their judgments were found to be correlated to their own perceived effort judgments.

### Study 2b

One way to interpret the results of the previous Study 2a is that people think of the costs and benefits for involved agents; they consider the utility of the epistemic action. One more variable in the expected utility calculation is the likelihood of the negative event happening. Study 2b investigates its relevance in the ascription of responsibility. Study 2b tests the hypothesis that, when ascribing responsibility in cases in which assessed agents bring about a bad outcome, people consider counterfactuals in which the expected value of the epistemic action is sufficiently high (H2b). This expected value is dependent on the perception of the probability that taking the epistemic action would lead to changing one’s mind about the best course of action. We manipulate such probability by changing the subjective prior probability that the bad outcome will follow from a given course of action (foreseeability of the event). For that purpose, we designed three different scenarios, and across these scenarios, we compared two conditions in which we manipulated the agent’s subjective prior probability of a negative outcome occurring: in the *Improbable* condition there is a low prior probability of the relevant event happening; in the *Probable* condition the prior probability of the relevant event happening is high. We measured participants’ responsibility judgments by prompting them to rate on a Likert scale to what extent they agreed that the agent was responsible for the bad outcome (Responsibility Question). We also measured their understanding of the prior probability by prompting them to rate on a Likert scale how likely they would have thought it was that the relevant event had happened (Probability Question).

We predicted that participants would more likely ascribe responsibility to agents who believed it was likely that their choice would lead to a negative outcome (*Probable* condition), than to those agents who believed it was very unlikely that their choice would lead to this outcome (*Improbable* condition). The former more than the latter are expected to check the information. In other words, agents who do not take the epistemic action are more likely to be judged responsible for the bad outcome when the epistemic action is likely to change the agent’s choices. We furthermore predicted that the lower the perceived likelihood of the relevant event happening, the less likely they will be judged responsible.

**Figure 5:**
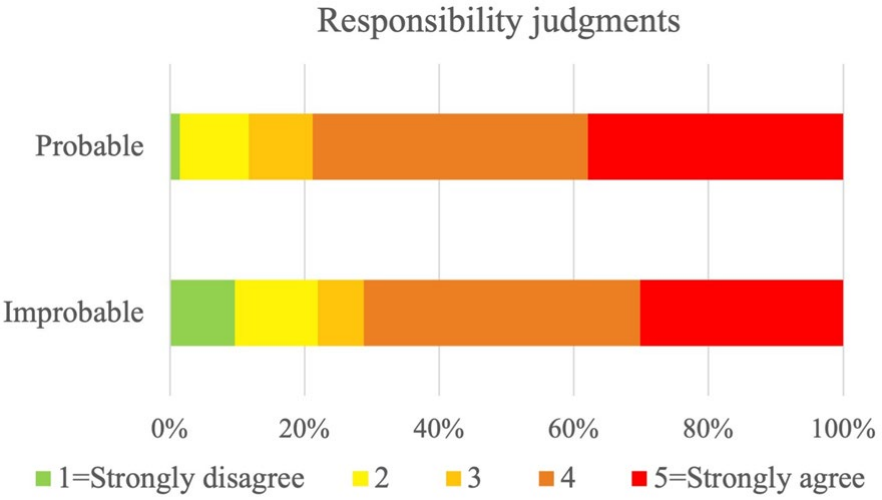
Distributions of responsibility judgments when the subjective prior probability of the relevant event happening is low and high. In the *Improbable* condition, participants read scenarios in which an agent’s subjective probability of the negative event happening was low. In the *Probable* condition, this subjective probability was high. Participants answered the question: “To what extent do you agree with the statement: ‘[Agent] is responsible for [outcome]’?”. Answers were given on a five-point scale from ‘Strongly disagree’, to ‘Strongly agree’. Responsibility ascribed was not statistically different across the two conditions.

To test H2b, we ran a cumulative link mixed model to assess the effect of the experimental condition on participants’ ratings. We compared our model with a null model using a likelihood ratio test which indicated that the cumulative link mixed model including condition did not provide a better fit for the data than that model without it $\chi ^{2}(1) = 2.32$, $p = 0.127$ (See Supplementary Table S8a online). Our prediction that the two conditions would differ was not confirmed. See Fig. 5.

Finally, we ran a Spearman rank correlation test. As predicted, the test showed a significant weak positive relationship between Responsibility judgments and Probability, $r_s (281) = 0.300$, $p < 0.001$.

Our findings did not confirm our hypotheses: although we spotted the trend that participants ascribed responsibility more often to agents who believed it was highly likely that their actions would produce negative outcomes, this result was not statistically significant.

On the other hand, Spearman’s correlation showed that participants’ responsibility judgments were related to their perception of the likelihood of the relevant event.

## Discussion

Our studies investigate how and when people ascribe responsibility to agents who did not predict the consequences of their actions. We hypothesized that this process involves counterfactual thinking, which includes thinking of alternative epistemic intentions. The results from three out of four of our studies corroborate this general hypothesis. They show that people are sensitive to information about agents’ epistemic states, actions, and intentions when ascribing responsibility.

Our findings show that agents who knowingly engaged in actions that caused a bad outcome are more often judged responsible than agents who did not know about these consequences; and that agents who had the opportunity to take an epistemic action to acquire relevant information were more often judged responsible compared to agents who did not have such opportunity (Study 1a). Furthermore, having prosocial epistemic intentions mitigates responsibility ascription, even when the epistemic action is not actually taken and when negative consequences still occur. By contrast, agents who do not want to learn about the risks of causing negative outcomes reveal their lack of concern for others. This allows assessors of responsibility to conceive a counterfactual where concern for others generates intentions that eventually cause a better outcome. Based on such counterfactual, assessors ascribe responsibility to agents who did not want to learn (Study 1b). Finally, we show that the perceived effort needed for the epistemic action can also mitigate responsibility ascription to ignorant agents. When the possibility of acquiring information was instead too costly, participants were less likely to hold agents responsible for bringing about a bad outcome (Study 2a). We intended to show that the expected value of an epistemic action would also be taken into account (Study 2b), but the data we gathered only showed a trend rather than strong evidence.

Overall, our studies provide evidence that the causal role of epistemic intentions is decisive for responsibility ascription, even though their causal role in bringing about the bad outcome is more distal than the choice that directly causes a bad outcome. Importantly, the beliefs that the agent held are themselves considered as resulting from epistemic actions (or lack thereof). Epistemic actions are, in turn, motivated by epistemic intentions which reveal how much concern for others the agent had. People do have expectations that agents act with some minimal concern for others. If a counterfactual shows that a bad outcome could have been avoided, had the agent been more concerned and thus motivated to check the risks of creating problems for others, then the agent is deemed responsible. Langenhoff et al.’s (2021) model of responsibility ascription holds that dispositional inference and causal attributions are assessed independently and then combined to determine whether the agent assessed is responsible or not^32^. By contrast, our model states that the cognitive processes of responsibility ascription involve assessing which intentions could have caused a better outcome for others. This reveals whether an agent sufficiently cared for others, or not. Our cognitive model is compatible with Alicke’s (2020) “culpable control” model in which negative character inferences influence blame by increasing the perception of the agent’s control over the outcome^33^. In our model, however, even agents who did not assert their control over the outcome can be excused if they can be perceived as showing sufficient care for others.

The results we presented are in line with recent findings that show that willfully ignorant agents are judged more harshly than those who did not suspect their choice could have bad consequences^34^. They are also compatible with findings that show that people are more likely to ascribe responsibility to agents who intentionally remained ignorant than to agents who did not suspect there was a risk to assess^3^. The authors explain the harsher moral judgments towards willfully ignorant agents by suggesting that people perceive their *mens rea* (guilty mind) and judge them to be more antisocial^34^. In our studies, we show that agents who suspect that a bad outcome might occur and yet do not intend to further assess the risks are judged as responsible as agents who know about the negative consequences their choice will have. This finding contrasts with some previous results where knowledgeable agents were found more culpable than willfully ignorant ones^34^. However, in our case, the dependent variable was responsibility, while Kirfel et al.’s (2023) study asked about culpability, which might be the source of the difference.

Our results show the relevance of the effort needed to learn the relevant information in responsibility ascription. This result is compatible with a previous study showing that the number of epistemic actions needed to get to the relevant information is a good predictor of causality ascription^35^. On the other hand, another study argues that the difficulty of performing the epistemic action does not excuse an agent who did not act to learn relevant information^34^. In our Study 2a, we manipulated one factor only: the effort needed. Our results suggest that it clearly is a factor for ascribing responsibility. This is best interpreted as evidence that people weigh the costs and benefits for the agent when they consider why they failed to take an epistemic action. In our experimental manipulations, the expected utility of acquiring relevant knowledge in these conditions is informative of the agent’s concern for others. If the cost of an epistemic action is low compared to others’ expected benefit, people infer that the agent who does not perform the epistemic action has little concern for others or low prosocial preferences^36^; by contrast, if the cost of the epistemic action is high compared to others’ expected benefit, then people cannot conclude that the agent does not have at least some minimal concern for others. The agent might have prosocial preferences, which have been outweighed by the cost of taking the epistemic action.

We hypothesized that the perceived likelihood that the bad outcome might occur would influence responsibility ascription, but we did not gather evidence for this hypothesis. We found a weak correlation between the measures of perceived likelihood and responsibility ascription, but we did not document a significant effect of our manipulation. Our prediction was motivated by the results of previous studies showing that foreseeability influences moral judgments^37,38^. Some more recent studies, however, do not show that the agent’s suspicion that the negative event could happen influences moral judgment^3,34^. It seems still premature to draw conclusions about people’s sensitivity to foreseeability in responsibility ascription. There are several possible explanations for the lack of significant results in our Study 2b. First, it is possible that our hypothesis does not stand and that people expect agents to take epistemic actions even when the action is not expected to be very informative. Second, the effect of likelihood may be more important when the consequences are less severe. When stakes are not negligible, such as in our stories, agents are expected to take actions that do not ask for significant sacrifice on their part. Third, our manipulation might not have been as strong as expected. While the perceived likelihood differed between the two conditions in our manipulation check (See Supplementary Table Sb online), absolute likelihood in the *Improbable* condition might have been overestimated. As a consequence, both conditions might have been perceived as expressing a high likelihood that the epistemic action would be informative.

Moral and legal philosophers have offered normative analyses on the responsibility and blameworthiness of willfully ignorant agents^39–41^. Our studies enable us to compare lay people’s intuitions with the normative explanation of when responsibility should be ascribed. According to philosophers’ normative accounts, agents are expected to get informed before acting. This duty is called procedural epistemic obligation^42^, or moral duty to get informed before acting^43^. On the one hand, agents are not expected to be informed about every possible situation. Wieland (2017) argues that not fulfilling the moral duty to get informed does not necessarily make a person morally blameworthy^41^. On the other hand, breaching the moral duty to get informed without justification qualifies as willful ignorance^44^ and is condemnable. The Quality of Will framework^27^ argues that if an agent showed moral concern in his actions, then they should be considered blameless; if they did not, they should be judged as blameworthy. Our studies show that laypeople’s intuitions are compatible with these philosophical accounts. When agents show moral concern, reflected in their intention to learn whether a choice risks hurting or causing problems to others, they are less often deemed responsible if a bad outcome happens nonetheless. Philosophers’ normative accounts also discuss an adequate threshold for the cost of epistemic action, above which the epistemic action is not expected anymore^41^. We show that laypeople think along these lines: high effort needed for the epistemic action mitigates responsibility ascription. People are sensitive to the sacrifice the assessed agent needs to make to get informed. Finally, the normative account considers foreseeability as a relevant factor in culpability judgments. According to the ‘comparative culpability principle’ (CCP)^45^, if all the other aspects are equal, the agent who was more confident that their action would produce some consequence should be more culpable. Participants in our study did not show sensitivity to this information, and so the topic needs to be investigated further.

## Methods

### Study 1a

The methods and statistical plan have been preregistered. You can find the preregistration document at: https://osf.io/6ysb9.

#### Participants

The initial preregistered analysis for this study was a non-parametric Kruskal-Wallis test and we therefore determined the sample size in view of that. More information can be found in the Supplementary Note online. A power analysis using the package ‘pwr’^46^ in R^29^ indicated that a total sample size of 128 participants (i.e. 384 data points) would detect a low effect size (f = 0.1) with a predicted statistical power of $86.8 \%$ using a one-way ANOVA with alpha at $0.05$. If the effect size is low-to-medium (f = 0.15), the predicted power increases to $99 \%$. Since we initially planned to run a non-parametric one-way ANOVA, given the ordinal nature of our data^47^, we added $\approx 15\%$ to our desired sample^48^. We thus planned to recruit 150 participants. We included the data from all participants who had already begun the experiment when we reached the desired number and closed the survey collector.

Participants were recruited from Prolific (www.prolific.com) and compensated at a rate of 9 GBP per hour of participation, evaluated by Prolific as a good payment. All participants were older than 18 and they all gave their informed consent to participate in the study. The only selection criterion was the use of English as primary, first, and fluent language. We collected data from 151 participants. As one participant took longer than expected to complete the survey, the recruitment platform reached the final sample ($N = 150$) before the participant completed their experimental session; we thus included them in our analysis.

Data was discarded in the case of participants who did not answer correctly the attention check question related to that scenario. If there was a mistake on only one or two scenarios out of three, only data points related to those scenarios were discarded. This resulted in excluding 25 data points, leaving 142 data points in the *Full Ignorance* condition, 144 in the *Willful Ignorance* condition, and 142 in the *Knowledge* condition. The experiment lasted roughly five minutes (Mdn = 5 min 4s). Here and elsewhere, the methods used were in accordance with the international ethical requirements of psychological research and were approved by the Psychological Research Ethics Board (PREBO, Ref: 2022/18) from the Central European University in Vienna for conducting the study.

#### Materials

As material for this study, we used nine vignettes describing some real-life social situations. We designed three different scenarios with three different conditions. Three different conditions of one scenario differ minimally: only the crucial manipulation part of the text is changed across conditions. All vignettes were of similar length and not longer than a few short paragraphs.

#### Procedure and design

We implemented our stimuli on the Qualtrics software^49^. Participants were instructed to read stories described in vignettes and answer a few questions. We designed three different scenarios and each of them occurred in three different conditions. We employed a between-subject design so that one participant saw only one condition of the scenario.

The scenarios follow a similar structure: an introduction describing the context with main character A doing B (general); information about epistemic states and actions that we manipulated depending on the experimental condition; main character doing S; and finally, consequences. To see an example of one scenario across the three conditions, see Table 1. Each participant randomly read the three stories, and they were presented always with all of the three conditions (one condition per story). Participants were assigned the scenarios in a randomized order. The three experimental conditions are:

1. *Knowledge* (i.e., Agent A acts knowingly); in this condition, the agent knows their action would produce a bad outcome, but they proceed with the behavior nevertheless;
2. *Willful Ignorance* (Agent A is willfully ignorant); in this condition, the agent does not know the relevant information, but has the opportunity to take a simple epistemic action to acquire such knowledge and willfully does not take it;
3. *Full Ignorance* (Agent A acting unknowingly); in this condition, the agent does not know the relevant information, nor the possibility of getting that information or a reason to search for it.

**Table 1:** Example of the three conditions of Study 1a in one of the scenarios.

Background		
Rose was on her way to her dentist appointment at 11 am. She went by car and parked in the only available spot.		
Full Ignorance	Willful Ignorance	Knowledge
The sign saying that spot is reserved was moved by someone. She didn’t know about the reservation and she parked there.	She saw there was a sign in front of the parking spot, saying whether and at what time during the day the spot is reserved. She didn’t stop to check what it said exactly, and she parked there.	She saw the sign saying the spot was reserved at that time. She parked there.
Outcome		
The sign indicates that the spot is reserved between 10am and 2pm for persons with disabilities. Consequently, a man couldn’t park anywhere and missed his weekly therapy.		

After reading the story participants first were prompted to evaluate the agent’s behavior. In addition to that, we asked them to elaborate on their answer. Then, on the next page, they received an attention check question in a multiple-choice form, to ensure they read the story carefully. This attention check was also used as an exclusion criterion. The evaluation of the agent’s behavior was done by ascription of responsibility, operationalized as a prompted judgment that answers the following question (Responsibility Question):

To what extent do you agree with the statement: ‘Agent A is responsible for B experiencing the consequence C’?

Participants were instructed to give answers on a 5-point Likert scale: ‘Strongly disagree’, ‘Somewhat disagree’, ‘Neither agree nor disagree’, ‘Somewhat agree’, and ‘Strongly agree’. For example: “To what extent do you agree with the statement: ‘Rose is responsible for the man missing his therapy’?”. Questions following each story were presented in a fixed order. It was important to present the Responsibility question and the Elaboration question immediately after the story, due to the possibility of attention check influencing the answer, although we made sure that the attention check questions were as neutral as possible. Moreover, we wanted to have the attention check on a separate page without the possibility to go back to the story. This way, we wanted to make sure participants were really paying attention to the story.

### Study 1b

The methods and statistical plan have been preregistered. You can find the preregistration document at: https://osf.io/u9w6k.

#### Participants

Following our previous studies, as well as Kirfel and Hannikainen (2022) and Kirfel, Bunk, and Gerstenberg (2023), we planned to collect the data from 450 participants, thus approximately 150 per condition, or 50 per scenario. We included data from all participants who had already begun the experiment when we reached the desired number and closed the survey collector.

Participants were recruited from Prolific (www.prolific.com) and compensated at a rate of 9 GBP per hour of participation, evaluated by Prolific as a good payment. All participants were older than 18 and they all gave their informed consent to participate in the study. The only selection criterion was the use of English as primary, first, and fluent language. We collected data from 452 participants. As two participants took longer than expected to complete the survey, the recruitment platform reached the final sample ($N = 450$) before the participant completed their experimental session; we thus included them in our analysis.

Data was discarded in the case of participants who did not answer correctly the attention check question related to that scenario. This resulted in excluding 27 data points, leaving 144 data points in the *Willful Ignorance* condition, 142 in the *Circumstantial Ignorance* condition, and 138 in the *Misinformed* condition. The experiment lasted roughly 2 min (Mdn = 1 min 37 s).

#### Materials

Material for this study were the same vignettes used in Study 1a, adjusted to include the manipulation required by Study 1b.

#### Procedure and design

The procedure was similar to the procedure followed in Study 1a. However, participants read only one scenario and they were asked to answer different additional questions. We used the same platforms for collecting the data (Qualtrics software) and recruiting participants (www.prolific.com) with the same payment rate set in Study 1a.

Participants’ task was to read a story, and after the story, they first got a question to evaluate the agent’s behavior, which was our dependent variable. Then, on the next page, they first got an attention check question and then a question to evaluate the perceived agent’s care to learn the relevant information. There were three different scenarios and each of them occurred in three different conditions. To see an example of one scenario across the two conditions, see Table 2. Each participant was randomly assigned to one of the three conditions of the one randomly selected scenario. The three experimental conditions are:

1. *Willful Ignorance* (i.e., Agent A has no prosocial epistemic intention and does not take the epistemic action); in this condition, the agent does not want to know if their action would produce the bad outcome, and they do not take the epistemic action to learn. They produce the negative consequence;
2. *Circumstantial Ignorance* (Agent A has a prosocial epistemic intention but does not take the epistemic action); in this condition, the agent wants to know if their action would produce the bad outcome, but the circumstances make it impossible for them to take the epistemic action to learn. They produce the negative consequence;
3. *Misinformed Ignorance* (Agent A has a prosocial intention and takes the epistemic action); in this condition, the agent wants to know if their action would produce the bad outcome and they take the epistemic action to learn, but due to someone else’s fault the relevant information was wrong. They produce the negative consequence.

**Table 2:** Example of all the three conditions of Study 1b in one of the scenarios.

Background		
Rose was on her way to her dentist appointment at 11 am. She went by car and parked in the only available spot.		
Willful Ignorance	Circumstantial Ignorance	Misinformed Ignorance
She saw there was a sign in front of the parking spot, saying whether and at what time of the day the spot is reserved. To check when exactly the spot is reserved, she would have needed to read the sign. She didn’t want to know whether the spot was reserved, and she didn’t read the sign; she parked there and went to her dentist appointment. The sign indicates that the spot is reserved between 10am and 2pm for persons with disabilities.	She saw there was a sign in front of the parking spot, saying whether and at what time of the day the spot is reserved. To check when exactly the spot is reserved, she would have needed to read the sign. She wanted to know whether the spot was reserved, but somebody took the sign from the pathway, so it was impossible for her to get the information. She parked there and went to her dentist appointment. The sign indicates that the spot is reserved between 10am and 2pm for persons with disabilities.	She saw there was a sign in front of the parking spot, saying whether and at what time of the day the spot is reserved. To check when exactly the spot is reserved, she needed to read the sign. She wanted to know whether the spot was reserved, so she read the sign and saw that it was not reserved at that time. She parked there and went to her dentist appointment. It turned out that the parking maintenance employee made a mistake and put the wrong information on the sign. The spot was actually reserved between 10am and 2pm for persons with disabilities.
Outcome		
Consequently, a man couldn’t park anywhere and missed his weekly therapy.		

Our main dependent variable is the ascription of responsibility, and it was operationalized in the same manner as in Study 1a. Another dependent variable is the perception of the agent’s care to learn the relevant information. It was operationalized through the following question (Care Question):

How much did Agent A care about learning information X?

Participants were instructed to give their answer on a 5-point Likert scale, where 1 is ’A didn’t care at all’ and 5 is ’A cared a lot’. For example, “How much did Rose care about whether the spot was reserved?” (Rose is Agent A, whether the spot was reserved is information X).

Between design was employed due to the possibility that answering the Care Question would influence the answers to the Responsibility question.

### Study 2a

The methods and statistical plan have been preregistered. You can find the preregistration document at: https://osf.io/bzcpq

#### Participants

The initial preregistered analysis for this study was a non-parametric Mann-Whitney t-test and we therefore determined the sample size in view of that. More information can be found in the Supplementary Note online. A power analysis using the package ‘pwr’^46^ in R^29^ indicated that a total sample size of 260 participants would detect a low effect size (d = 0.2) with a predicted statistical power of $62.4\%$ using an independent-measures t-test with alpha at $.05$. If the effect size is low-to-medium ($d = 0.35$), the predicted power decreases to $97.8 \%$. Since we planned to run a non-parametric test, given the nature of our data^47^, we added $\approx 15\%$ to our desired sample^48^. From this analysis, we aimed to collect data from 300 participants. We included the data from all participants who had already begun the experiment when we reached the desired number and closed the survey collector.

Participants were recruited from Prolific (www.prolific.com) and compensated at a rate of 9 GBP per hour of participation, evaluated by Prolific as a good payment. All participants were older than 18 and they all gave their informed consent to participate in the study. The only selection criterion was the use of English as primary, first, and fluent language. We collected the data from 308 participants, since the collector stops when the number of participants who concluded the study is 300, but discards participants who started the study but gave up before the final submission. Since Qualtrics counts in all the data points that started the questionnaire, and due to the fact that we did not collect participants’ IDs, we could not distinguish which participants gave up on Prolific in order to exclude them. After the exclusion of the data points that were not complete (answering none or only one question out of three related to the scenario), a total of 306 data points were taken into consideration. The exclusion criterion was that the data points of participants who did not answer correctly on an attention check would not be used. Since no participant made a mistake, all 306 data points were used in further analyses.

Data was discarded in the case of participants who did not answer correctly the attention check question related to that scenario. This resulted in excluding 13 data points, leaving 146 data points in the *Low Effort* condition and 147 in the *High Effort* condition. The experiment lasted roughly two minutes (Mdn = 1 min 34s).

#### Materials

Material for this study were the same vignettes from Study 1a and Study 1b adjusted to include the manipulation required by Study 2a.

#### Procedure and design

The procedure was similar to the procedure followed in Study 1b. However, participants were asked to answer different additional questions. We used the same platforms for collecting the data (Qualtrics software) and recruiting participants (www.prolific.com) with the same payment rate set in the previous studies.

Participants’ task was to read a story, and after the story, they first got a question to evaluate the agent’s behavior, which was our dependent variable. Then, on the next page, they first got an attention check question and then a question to evaluate the perceived effort needed for the epistemic action. There were three different scenarios and each of them occurred in two different conditions. To see an example of one scenario across the two conditions, see Table 3. Each participant was randomly assigned to one of the two conditions of the one randomly selected scenario. Two conditions are:

1. *Low Effort* (Low cost of the epistemic action): in this condition agent would need to do the simple action and put a little effort in order to acquire the relevant knowledge about the consequences of their action;
2. *High Effort* (High cost of the epistemic action): in this condition agent would need to put a lot of effort in order to acquire the relevant knowledge about the consequences of their action.

**Table 3:** Example of the two conditions of Study 2a in one of the scenarios.

Background	
Rose was on her way to her dentist appointment at 11 am. She went by car and parked in the only available spot.	
Low Effort	High Effort
She saw there was a sign in front of the parking spot, saying whether and at what time of the day the spot is reserved. To check when exactly the spot is reserved, she would have needed to read the sign. She didn’t do so; she parked there and went to her dentist appointment.	She saw there was a sign in front of the parking spot, saying that you should check with the parking attendant whether the spot is reserved. The attendant was on a lunch break, so to check when exactly the spot is reserved she would have needed to wait for him to come back at some point. She didn’t do so; she parked there and went to her dentist appointment.
Outcome	
The sign indicates that the spot is reserved between 10am and 2pm for persons with disabilities. Consequently, a man couldn’t park anywhere and missed his weekly therapy.	

Our main dependent variable is the ascription of responsibility, and it was operationalized in the same manner as in Study 1. Another dependent variable is the perception of effort needed to initiate the epistemic action. It was operationalized through the following question (Perceived Effort Question):

How difficult would have been for agent X to check the information W?

Participants were instructed to give answers on a 5-point Likert scale: ’Very easy’, ’Somewhat easy’, ’Neither easy nor difficult’, ’Somewhat difficult’, and ’Very difficult.’ For example: “How difficult would have been for Rose to check whether the spot was reserved?” (Rose is the agent, information about whether the spot is reserved).

We employed a between-subjects design due to the possibility that answering the Perceived Effort question would influence the answers to the subsequent Responsibility Question.

### Study 2b

The methods and statistical plan have been preregistered. You can find the preregistration document at: https://osf.io/qxphg.

#### Participants

Following our previous studies, as well as Kirfel and Hannikainen (2022) and Kirfel, Bunk, and Gerstenberg (2023), we planned to collect the data from 300 participants, thus approximately 150 per condition, or 50 per scenario. We included data from all participants who had already begun the experiment when we reached the desired number and closed the survey collector.

Participants were recruited from Prolific (www.prolific.com) and compensated at a rate of 9 GBP per hour of participation, evaluated by Prolific as a good payment. All participants were older than 18 and they all gave their informed consent to participate in the study. The only selection criterion was the use of English as primary, first, and fluent language. We collected data from 303 participants. As three participants took longer than expected to complete the survey, the recruitment platform reached the final sample ($N = 300$) before the participant completed their experimental session; we thus included them in our analysis.

Data was discarded in case of participants who did not answer correctly the attention check question related to that scenario. This resulted in excluding 20 data points, leaving 146 data points in the *Improbable* condition and 137 in the *Probable* condition. The experiment lasted roughly two minutes (Mdn = 1 min 39s).

#### Materials

Material for this study were two out of three vignettes from Study 1a, Study 1b, and Study 2a. The third vignette was a new one created for this study because one of the vignettes previously used could not be updated adequately for the manipulation in this study. All vignettes were adjusted to include the manipulation required by Study 2b.

#### Procedure and design

The procedure was similar to the procedure followed in Study 1b and Study 2a. However, participants were asked to answer different additional questions. We used the same platforms for collecting the data (Qualtrics software) and recruiting participants (www.prolific.com) with the payment rate same as in the previous studies.

Participants’ task was to read a story, and after the story, they first got a question to evaluate the agent’s behavior, which was our dependent variable. Then, on the next page, they first got an attention check question and then a question to evaluate the perceived likelihood of the relevant event happening. There were three different scenarios and each of them occurred in two different conditions. To see an example of one scenario across the two conditions, see Table 4. Each participant was randomly assigned to one of the two conditions of the one randomly selected scenario. The two experimental conditions are:

1. *Improbable* (Low probability of the relevant event happening): in this condition the subjective probability of the relevant event happening is low since in the agent’s experience it happens very rarely or never;
2. *Probable* (High probability of the relevant event happening): in this condition the subjective probability of the relevant event happening is high since in the agent’s experience it happens very often or always.

**Table 4:** Example of the two conditions of Study 2b in one of the scenarios.

Background	
Rose was on her way to her dentist’s appointment at noon. She went by car and parked in the only available spot.	
Improbable	Probable
She saw there was a sign in front of the parking spot, saying that you should check with the parking attendant whether the spot is reserved. This sign had been there for a year, and, in her experience, that spot was actually never reserved. She didn’t check with the parking attendant whether the spot was reserved; she parked there and went to her dentist appointment.	She saw there was a sign in front of the parking spot, saying that you should check with the parking attendant whether the spot is reserved. This sign had been there for a year, and, in her experience, that spot was reserved in 90% of the cases. She didn’t check with the parking attendant whether the spot was reserved; she parked there and went to her dentist appointment.
Outcome	
As it happens, the spot was reserved at that time for people with disabilities. Consequently, a man couldn’t park anywhere and missed his weekly therapy.	

Our main dependent variable is the ascription of responsibility, and it was operationalized in the same manner as in the previous studies. Another dependent variable is the perceived likelihood of the relevant events. Relevant events are those that participate in causing negative consequences for other people. This likelihood can be understood as a proxy for the  agent’s beliefs about what would happen. It is operationalized through the following question (Probability Question):

How likely would you have thought it was that relevant event X had happened?

Participants are instructed to indicate the probability by using the slider, where 0 is ’Improbable’ and 100 is ’Probable’. For example: “How likely would you have thought it was that Karl had to work on that weekend?” (Karl working on that weekend is the relevant event X).

Between design was employed due to the possibility that answering the Probability question would influence the answers to the Responsibility Question.

### Supplementary Information

Supplementary Information.

## Data Availability

The data of Study 1a, Study 1b, Study 2a, and Study 2b is uploaded on https://osf.io/y9gxj/.
