# Supplementary material for: The importance of epistemic intentions in ascription of responsibility
Source: Sci Rep. 2024 Jan 12;14:1183. doi: 10.1038/s41598-023-50961-0 (PMC10786917; doi:10.1038/s41598-023-50961-0)
Supplement: Supplementary file 1 — Supplementary Information. [file 41598_2023_50961_MOESM1_ESM.pdf]

Supplementary Information for: *The importance of epistemic intentions in ascription of responsibility*

Katarina M. Kovacevic[1], Francesca Bonalumi[1,2], and Christophe Heintz[1]

[1]Central European University, Department of Cognitive Science, Vienna, 1100, Austria

[2]School of Social Sciences and Technology, Technical University Munich, Munich, Germany

Table of Contents

DESCRIPTIVE STATISTICS FOR TWO DEPENDENT VARIABLES ACROSS CONDITIONS AND SCENARIOS 2

SUPPLEMENTARY TABLE S1A 2
SUPPLEMENTARY TABLE S1B 2
SUPPLEMENTARY TABLE S2A 3
SUPPLEMENTARY TABLE S2B 3
SUPPLEMENTARY TABLE S2C 4
SUPPLEMENTARY TABLE S3A 4
SUPPLEMENTARY TABLE S3B 4
SUPPLEMENTARY TABLE S3C 5
SUPPLEMENTARY TABLE S4A 5
SUPPLEMENTARY TABLE S4B 5
SUPPLEMENTARY TABLE S4C 6

DETAILS OF ANALYSES PRESENTED IN THE MANUSCRIPT 6

SUPPLEMENTARY TABLE S5A 6
SUPPLEMENTARY TABLE S5B 7
SUPPLEMENTARY TABLE S5C 7
SUPPLEMENTARY TABLE S6A 8
SUPPLEMENTARY TABLE S6B 8
SUPPLEMENTARY TABLE S6C 8
SUPPLEMENTARY TABLE S7A 9
SUPPLEMENTARY TABLE S7B 9
SUPPLEMENTARY TABLE S8A 9

DEVIATIONS FROM PREREGISTRATION 10

SUPPLEMENTARY NOTE 10

PREREGISTERED ANALYSES 10

SUPPLEMENTARY TABLE S9A 10
SUPPLEMENTARY TABLE S9B 10
SUPPLEMENTARY TABLE S10 11

R DATA ANALYSES CODES 12

DESCRIPTIVE STATISTICS FOR TWO DEPENDENT VARIABLES ACROSS CONDITIONS AND SCENARIOS

Descriptive analyses were done using Jamovi 2.3[1] and JASP 0.15[2].

Study 1a

Supplementary Table S1a

Descriptives - Responsibility judgments. The question was "To what extent do you agree with the statement: "Agent A is responsible for Agent B facing consequences C." Answers were coded from 1 (Strongly disagree) to 5 (Strongly agree).

Condition	Scenario	*Mean*	*Sd*	N
Full Ignorance	Money in the street	2.265	1.114	49
	Parking spot	1.690	1.179	42
	Phone	1.529	1.065	51
Willful Ignorance	Money in the street	3.204	1.323	49
	Parking spot	4.170	1.257	47
	Phone	3.625	1.214	48
Knowledge	Money in the street	3.900	1.233	50
	Parking spot	4.047	1.112	43
	Phone	4.408	0.762	49

Supplementary Table S1b

Distribution of Responsibility judgments across three conditions. The question was "To what extent do you agree with the statement: "Agent A is responsible for Agent B facing consequences C."

Condition	Strongly disagree	Disagree	Neither agree nor disagree	Agree	Strongly agree
Full Ignorance	46	40	6	14	6
Willful Ignorance	13	22	13	49	47
Knowledge	7	7	10	56	62

Study 1b

Supplementary Table S2a

Descriptives- Care judgments: The question was "How much did agent A care about learning information X?". Answers were coded from 1 (A didn't care at all) to 5 (A cared a lot).

Condition	Scenario	*Mean*	*Sd*	N
Circumstantial Ignorance	Money in the street	4.000	0.770	55
	Parking spot	3.043	0.815	46
	Phone	3.171	1.022	41
Misinformed Ignorance	Money in the street	4.385	1.013	52
	Parking spot	4.237	1.076	38
	Phone	3.833	0.907	48
Willful Ignorance	Money in the street	1.537	0.809	41
	Parking spot	1.213	0.463	47
	Phone	1.446	0.685	56

Supplementary Table S2b

Descriptives – Responsibility judgments. The question was "To what extent do you agree with the statement: "Agent A is responsible for Agent B facing consequences C." Answers were coded from 1 (Strongly disagree) to 5 (Strongly agree).

Condition	Scenario	*Mean*	*Sd*	N
Willful Ignorance	Parking spot	4.250	1.101	48
	Money in the street	3.195	1.289	41
	Phone	3.768	1.175	56
Circumstantial Ignorance	Parking spot	2.652	1.251	46
	Money in the street	1.891	0.975	55
	Phone	2.488	1.227	41
Misinformed Ignorance	Parking spot	1.658	1.169	38
	Money in the street	1.481	0.896	52
	Phone	1.667	0.808	48

[1] The jamovi project. *jamovi* (version 2.3) [Computer Software] Retrieved from https://www.jamovi.org (2022).
[2] JASP Team. JASP (Version 0.15) [Computer software] (2021).

Supplementary Table S2c

Distribution of Responsibility judgments across three conditions. The question was "To what extent do you agree with the statement: "Agent A is responsible for Agent B facing consequences C."

Condition	Strongly disagree	Disagree	Neither agree nor disagree	Agree	Strongly agree
Willful Ignorance	10	21	10	56	48
Circumstantial Ignorance	43	49	17	29	4
Misinformed Ignorance	85	37	6	7	3

Study 2a

Supplementary Table S3a

Descriptives - Responsibility judgments. The question was "To what extent do you agree with the statement: "Agent A is responsible for Agent B facing consequences C." Answers were coded from 1 (Strongly disagree) to 5 (Strongly agree).

Condition	Scenario	*Mean*	*SD*	N
Low Effort	Money in the street	3.417	1.217	48
	Parking spot	4.118	0.952	51
	Phone	3.894	0.938	47
High Effort	Money in the street	3.098	1.204	51
	Parking spot	3.620	1.244	50
	Phone	3.391	1.325	46

Supplementary Table S3b

*Descriptives - Difficulty judgments. The question was "How difficult would have been for agent X to check the information W?". Answers were coded from 1 (V*ery easy*) to 5 (Very difficult).*

Condition	Scenario	*Mean*	*SD*	N
Low Effort	Money in the street	1.521	0.618	48
	Parking spot	1.235	0.428	51
	Phone	1.340	0.700	47
High Effort	Money in the street	3.588	1.004	51
	Parking spot	3.700	0.931	50
	Phone	4.000	0.966	46

Supplementary Table S3c

Distribution of Responsibility judgments across three conditions. The question was "To what extent do you agree with the statement: "Agent A is responsible for Agent B facing consequences C."

Condition	Strongly disagree	Disagree	Neither agree nor disagree	Agree	Strongly agree
Low Effort	4	22	10	71	39
High Effort	13	34	13	60	27

Study 2b

Supplementary Table S4a

Descriptives - Responsibility judgments. The question was "To what extent do you agree with the statement: "Agent A is responsible for Agent B facing consequences C." Answers were coded from 1 (Strongly disagree) to 5 (Strongly agree).

Condition	Scenario	*Mean*	*Sd*	N
Probable	Parking spot	4.510	0.649	49
	Money in the street	3.073	1.081	41
	Taking the car	4.383	0.610	47
Improbable	Parking spot	4.327	0.899	49
	Money in the street	3.000	1.430	47
	Taking the car	3.740	1.139	50

Supplementary Table S4b

Descriptives – Probability judgments: The question was "How likely would you have thought it was that relevant event X had happened?". Answers were given on a slider from 0 (Improbable) to 100 (Probable).

Condition	Scenario	*Mean*	*Sd*	N
Probable	Parking spot	81.551	20.645	49
	Money in the street	77.927	21.002	41
	Taking the car	77.787	18.902	47
Improbable	Parking spot	51.469	22.595	49
	Money in the street	34.255	21.951	47
	Taking the car	29.940	25.263	50

Supplementary Table S4c

Distribution of Responsibility judgments across three conditions. The question was "To what extent do you agree with the statement: "Agent A is responsible for Agent B facing consequences C." :

Condition	Strongly disagree	Disagree	Neither agree nor disagree	Agree	Strongly agree
Probable	2	14	13	56	52
Improbable	14	18	10	60	44

DETAILS OF ANALYSES PRESENTED IN THE MANUSCRIPT

Analyses presented in the Manuscript have been done using R version 4.2.3[3] and the package 'ordinal'[4]. Codes for the analyses are given at the end of the document.

Study 1a

Prediction: Effect of condition on Responsibility

Null model: ratings ~ 1 + (condition|scenario) + (1|ID)
Full model: ratings ~ condition + (condition|scenario) + (1|ID)

Supplementary Table S5a

Likelihood ratio tests of linear link models:

	No. par	AIC	LogLik	LR.stat	*df*	*p*
Null model	11	1108.1	-543.04			
Full model	12	1103.9	-538.93	8.2168	2	.01643*

* $p < .05$, ** $p < .01$, *** $p < .001$

[3] R Core Team. R: *A Language and Environment for Statistical Computing.* R Foundation for Statistical Computing, Vienna, Austria (2022).
[4] Christensen, R. H. B. ordinal—regression models for ordinal data. R package version 2022.11-16 (2022).

Supplementary Table S5b

Summary of the model:

	Estimate	*SE*	*Z value*	*p*	*CI*
Willful Ignorance	3.046	0.793	3.839	.000124 ***	1.49 4.60
Knowledge	3.722	0.623	5.978	2.26e-09 ***	2.50 4.94
1|2	0.238	0.433	0.548		-0.61 1.09
2|3	1.621	0.449	3.609		0.74 2.50
3|4	2.125	0.458	4.642		1.23 3.02
4|5	3.932	0.495	7.943		2.96 4.90

Supplementary Table S5c

Pairwise comparisons:

Contrast	*Estimate*	*SE*	*Z value*	*p*
Full Ignorance – Willful Ignorance	-3.046	0.793	-3.839	.0004 **
Full Ignorance – Knowledge	-3.722	0.623	-5.978	< 0.0001 ***
Willful Ignorance – Knowledge	-0.677	0.472	-1.434	.4543

Study 1b

Prediction: Effect of condition on Responsibility

Null model: ratings ~ 1 + (condition|scenario)
Full model: ratings ~ condition + (condition|scenario)

Supplementary Table S6a

Likelihood ratio tests of linear link models:

	No. par	*AIC*	*LogLik*	*LR.stat*	*df*	*p*
Null model	10	1095.7	-537.83			
Full model	12	1090.0	-533.01	9.6394	2	0.00807 **

Supplementary Table S6b

Summary of the model:

	Estimate	*SE*	*Z value*	*p*	*CI*
Circumstantial I	-2.148	0.302	-7.118	1.09e-012 ***	-2.74 -1.56
Misinformed I	-3.505	0.461	-7.609	2.76e-14 ***	-4.41 -2.60
1|2	-3.035	0.487	-6.235		-3.99 -2.08
2|3	-1.525	0.472	-3.235		-2.45 -0.60
3|4	-1.004	0.467	-2.148		-1.92 -0.09
4|5	0.878	0.466	1.887		-0.03 1.79

Supplementary Table S6c

Pairwise comparisons:

Contrast	*Estimate*	*SE*	*Z value*	*p*
Willful I – Circumstantial I	2.15	0.302	7.118	< 0.0001 ***
Willful I – Misinformed I	3.50	0.461	7.609	< 0.0001 ***
Circumstantial I – Misinformed I	1.36	0.303	4.474	< 0.0001 ***

Study 2a

Prediction: Effect of condition on Responsibility

Null model: ratings ~ 1 + (1 + condition|scenario)
Full model: ratings ~ condition + (condition|scenario)

Supplementary Table S7a

Likelihood ratio tests of linear link models:

	No. par	*AIC*	*LogLik*	*LR.stat*	*df*	*p*
Null model	7	809.28	-397.64			
Full model	8	806.09	-366.04	5,1928	1	.0226*

Supplementary Table S7b

Summary of the model:

	Estimate	*SE*	*Z value*	*p*	*CI*
Low Effort	-0.652	0.227	-2.879	.00398 **	-1.10 -0.21
1|2	-3.217	0.375	-8.574		-3.95 -2.48
2|3	-1.487	0.306	-4.855		-2.09 -0.89
3|4	-1.083	0.300	-3.606		-1.67 -0.49
4|5	0.986	0.299	3.294		0.40 1.57

Study 2b

Prediction: Effect of condition on Responsibility

Null model: ratings ~ 1 + (1 + condition|scenario)
Full model: ratings ~ condition + (condition|scenario)

Supplementary Table S8a

Likelihood ratio tests of linear link models:

	No. par	AIC	LogLik	LR.stat	*df*	*P*
Null model	7	710.62	-348.31			
Full model	8	710.29	-347.15	2.3254	1	0.1273

DEVIATIONS FROM PREREGISTRATION

Supplementary Note

Justification for using Cumulative Link Mixed Models

In our pre-registration template for Study 1a and Study 2a, we have indicated non-parametric statistic to analyze our data, using Kruskal-Wallis and Mann-Whitney test, respectively. We specified the Responsibility judgment as a dependent variable, and Condition as an independent variable. Analyses were done using Jamovi 2.3.

After the feedback from reviewers, we decided to change the analysis to Cumulative Link Mixed Model (Christensen & Christensen, 2015). We opted for a mixed model so to acknowledge the fact we used multiple different vignettes in our experiments; and additionally, a cumulative model is appropriate for ordinal measures. Thus, our model included condition as fixed effect, and condition nested within vignettes as random effect.

Employing a new analysis plan slightly changed the results of Study 1a. Previous results provided by Kruskal-Wallis test and pairwise comparisons showed that Willful Ignorance and Knowledge conditions differed significantly (See Supplementary table S9b, page 10). The new analysis that included condition nested within vignettes as random effect did not show any difference.

PREREGISTERED ANALYSES

Study 1a

To analyze the results from Study 1a we previously preregistered a non-parametric Kruskal-Wallis test, since the measures were ordinal and our independent variable had three levels.

Supplementary Table S9a

Kruskal-Wallis Test

	χ^2	*df*	*p*	ε^2
Responsibility	163	2	< .001	.383

Supplementary Table S9b

Pairwise comparisons

		W	*p*
Full Ignorance	Willful Ignorance	14.13	< .001
Full Ignorance	Knowledge	16.61	< .001
Willful Ignorance	Knowledge	4.14	.010

Study 2a

To analyze the results from Study 2a we previously preregistered a non-parametric Mann-Whitney t-test, since the measures were ordinal and independent variable had two levels.

Supplementary Table S10

Independent Samples T-Test

	U	*p*	Rank-Biserial Correlation
Responsibility	8645	.002	.194

R DATA ANALYSES CODES

```
## clear out the environment
rm(list=ls())

#load packages
library(ordinal)
library(readxl)
library(emmeans)

#dataset
Results1 <- read_excel("Final_S1a.xlsx")
Results1b <- read_excel("Final_S1b.xlsx")
Results2 <- read_excel("Final_S2a.xlsx")
Results2b <- read_excel("Final_S2b.xlsx")
```

Study 1a

```
#factorise
head(Results1)
Results1$responsibility<-as.factor(Results1$responsibility)
Results1$scenario <- as.factor(Results1$scenario)
Results1$condition <- as.factor(Results1$condition)

## models
#build full model
s1m1 <- clmm(responsibility ~ condition + (condition|scenario) + (1|ID), data=Results1)
#build null model
s1m0 <- clmm(responsibility ~ 1 + (condition|scenario) + (1|ID), data = Results1)
#compare models
anova(s1m1, s1m0)

# LRT for p-values
drop1(s1m1, test = "Chisq")

confint(s1m1, level = 0.95)
summary(s1m1)

#pairwise comparisons
emm <- emmeans(s1m1, ~ condition)
contrast(emm, method = "pairwise", adjust = "bonferroni")
#P value adjustment: bonferroni method for 3 tests

#tranform logodds
exp(3.046)/(1+exp(3.046))
exp(-3.722)/(1+exp(-3.722))
exp(-0.677)/(1+exp(-0.6777))
```

Study 1b

```
#factorise
head(Results1b)
Results1b$responsibility<-as.factor(Results1b$responsibility)
Results1b$scenario <- as.factor(Results1b$scenario)
Results1b$condition <- as.factor(Results1b$condition)
Results1b$care <- as.factor(Results1b$care)

## models
#build full model
s1bm1 <- clmm(responsibility ~ condition + (condition|scenario), data=Results1b)
#build null model
s1bm0 <- clmm(responsibility ~ 1 + (condition|scenario), data = Results1b)

#compare models
anova(s1bm1, s1bm0)
# LRT for p-values
drop1(s1bm1, test = "Chisq")
confint(s1bm1, level = 0.95)
summary(s1bm1)
logit <- exp(s1bm1$coefficients)

#pairwise comparisons
emm <- emmeans(s1bm1, ~ condition)
contrast(emm, method = "pairwise", adjust = "bonferroni")

#transforms logodds
exp(3.505)/(1+exp(3.505))
```

Study 2a

```
#factorise S2
head(Results2)
table(Results2$responsibility)
table(Results2$responsibility, Results2$condition)
table(Results2$difficulty, Results2$condition)
Results2$responsibility<-as.factor(Results2$responsibility)
Results2$scenario <- as.factor(Results2$scenario)
Results2$condition <- as.factor(Results2$condition)
Results2$difficulty <- as.factor(Results2$difficulty)

## models
#build full model
s2m1 <- clmm(responsibility ~ 1 + condition + (1 + condition|scenario), data=Results2)
#build null model
s2m0 <- clmm(responsibility ~ 1 + (1 + condition|scenario), data = Results2)

#compare models
anova(s2m1, s2m0)

# LRT for p-values
drop1(s2m1, test = "Chisq")

confint(s2m1, level = 0.95)
summary(s2m1)
```

Study 2b

```
#factorise S2b
head(Results2b)
table(Results2b$resp)
table(Results2b$resp, Results2b$condition)
Results2b$resp<-as.factor(Results2b$resp)
Results2b$scenario <- as.factor(Results2b$scenario)
Results2b$condition <- as.factor(Results2b$condition)
Results2b$prob <- as.numeric(Results2b$prob)

mean(Results2b$prob[Results2b$condition == "1"], na.rm = TRUE)
mean(Results2b$prob[Results2b$condition == "2"], na.rm = TRUE)

data_1 <- Results2b[Results2b$scenario == "1", ]
data_2 <- Results2b[Results2b$scenario == "2", ]
data_3 <- Results2b[Results2b$scenario == "3", ]

## models
#build full model
s2bm1 <- clmm(resp ~ 1 + condition + (1 + condition|scenario), data=Results2b)
#build null model
s2bm0 <- clmm(resp ~ 1 + (1 + condition|scenario), data = Results2b)

#compare models
anova(s2bm1, s2bm0)
```
